# Intraosseous Temperature Change during Installation of Dental Implants with Two Different Surfaces and Different Drilling Protocols: An In Vivo Study in Sheep

**DOI:** 10.3390/jcm8081198

**Published:** 2019-08-11

**Authors:** Michele Stocchero, Yohei Jinno, Marco Toia, Marianne Ahmad, Evaggelia Papia, Satoshi Yamaguchi, Jonas P. Becktor

**Affiliations:** 1 Department of Oral & Maxillofacial Surgery and Oral Medicine, Faculty of Odontology, Malmö University, 20506 Malmö, Sweden; 2 Department of Materials Science and Technology, Faculty of Odontology, Malmö University, 20506 Malmö, Sweden; 3 Department of Biomaterials Science, Osaka University Graduate School of Dentistry, Osaka 565-0871, Japan

**Keywords:** oral implants, osseointegration, implant installation, anchorage technique, histology, osteotomy, intraosseous temperature, in vivo study, finite element model

## Abstract

Background: The intraosseous temperature during implant installation has never been evaluated in an in vivo controlled setup. The aims were to investigate the influence of a drilling protocol and implant surface on the intraosseous temperature during implant installation, to evaluate the influence of temperature increase on osseointegration and to calculate the heat distribution in cortical bone. Methods: Forty Brånemark implants were installed into the metatarsal bone of Finnish Dorset crossbred sheep according to two different drilling protocols (undersized/non-undersized) and two surfaces (moderately rough/turned). The intraosseous temperature was recorded, and Finite Element Model (FEM) was generated to understand the thermal behavior. Non-decalcified histology was carried out after five weeks of healing. The following osseointegration parameters were calculated: Bone-to-implant contact (BIC), Bone Area Fraction Occupancy (BAFO), and Bone Area Fraction Occupancy up to 1.5 mm (BA1.5). A multiple regression model was used to identify the influencing variables on the histomorphometric parameters. Results: The temperature was affected by the drilling protocol, while no influence was demonstrated by the implant surface. BIC was positively influenced by the undersized drilling protocol and rough surface, BAFO was negatively influenced by the temperature rise, and BA1.5 was negatively influenced by the undersized drilling protocol. FEM showed that the temperature at the implant interface might exceed the limit for bone necrosis. Conclusion: The intraosseous temperature is greatly increased by an undersized drilling protocol but not from the implant surface. The temperature increase negatively affects the bone healing in the proximity of the implant. The undersized drilling protocol for Brånemark implant systems increases the amount of bone at the interface, but it negatively impacts the bone far from the implant.

## 1. Introduction

Despite the high success rate of dental implants [1,2], early and late implant failures are still encountered [3,4]. It is known that the surgical approach is one key-factor for successful implant treatment [5]. It was advocated that an optimal surgical technique should provide initial mechanical implant stability necessary for the initiation of the osseointegration process [6,7]. Simultaneously, implants should be installed with a gentle and atraumatic surgical technique, avoiding excessive biomechanical and thermal stresses to the bone [8].

Undersized drilling is one of the most common surgical techniques for increasing primary implant stability. With this technique, an implant is installed in a substantially smaller osteotomy than its diameter [9]. The rationale of such a procedure is to maximize the initial implant contact with the bone locally and, thus, secure the implant stability [10]. This approach increases the implant rotational resistance, measured as the insertion torque value (ITV) [11]. This condition is often considered desirable [12], especially when immediate or early loading protocols are applied [13].

However, there are some concerns among researchers and clinicians regarding the host bone reaction to increased lateral compression, especially when the cortical bone layer is involved [14]. Excessive stresses and strains beyond bone physiological limits can have disadvantageous effects on the local microcirculation and bone cellular responses, leading to so-called bone compression necrosis [15]. In vivo studies and clinical research reported an extensive area of apoptotic osteocytes, tissue damage, and ultimately peri-implant bone loss [16,17,18]. Moreover, previous consensus reports indicated compression necrosis as a possible risk factor for peri-implant tissues disease [19,20].

Besides the over-compression of the pristine bone, a thermal injury may be an additional factor inducing bone necrosis. When the bone is heated above 53 °C, irreversible tissue damage was observed [21], while 47 °C for 1 min is considered as the border condition for the occurrence of an injury [22]. Although the risk of bone overheating during the drilling procedure was extensively investigated [23], the increase of temperature during implant installation, was seldom reported [24]. During the seating into an osteotomy site, energy is supplied to the bone and part of it is transferred as frictional heat [25]. According to thermodynamics, thermal energy is partially absorbed and conducted by the implant itself, and by the bone, in the form of temperature increase [26].

It is unknown whether the frictional heat generated during implant installation can cause an increase in bone temperature exceeding the threshold of initiating irreversible tissue damage. The temperature may be influenced by several factors, including implant-osteotomy size discrepancy and implant micro-design. One could postulate that the installation of an implant in an undersized osteotomy and the use of a moderately rough surface would generate a greater temperature increase in the bone than a larger osteotomy and a turned surface.

However, the in vivo intraosseous temperature during implant installation was never previously evaluated in a controlled experimental setting. Besides that, the effect of such temperature on histomorphometric parameters is unknown.

The primary aim of this in vivo study was to investigate the influence of the drilling protocol and implant surface on the intraosseous temperature change during dental implant installation. The secondary aims were to evaluate the influence of the temperature on osseointegration and peri-implant bone healing and to calculate the heat distribution in cortical bone.

## 2. Materials and Methods

### 2.1. Study Design and Samples

This study was approved by the ethical committee of the Ecole Nationale Vétérinaire d’Alfort, Paris, France, (reference number: 02343.03) and it is reported according to the ARRIVE (Animal Research Reporting of in Vivo Experiments) guidelines [27]. Ten female Finnish Dorset crossbred sheep (average; years old and 54 kg) were used and housed together for one week before surgery. A total of forty 3.75 mm × 7 mm dental implants (Brånemark system^®^ MKIII RP implant, Nobel Biocare AB, Göteborg, Sweden) were installed, including 20 implants with moderately rough oxidized titanium surface (TiUnite^®^ surface) and 20 implants with a turned surface. The present implant surfaces, investigated by a broad variety of preclinical and clinical research [28,29,30], presents the following surface characteristics according to Wennerberg and Albrektsson with the parameters Sa (the arithmetic average height deviation from mean plane) and Sdr (the developed surface ratio): 1.1 µm and 0.9 µm (Sa); 37% and 34% (Sdr) for TiUnite^®^ and turned surface, respectively [31].

### 2.2. Surgical Procedures

All surgeries were done under general anesthesia with ketamine (Imalgene 1000®, Merial, Villeurbanne, France), and diazepam (Valium, Roche, Boulogne-Billancourt, France) for injection, and with 2.5% isoflurane (Forane®/Forene®, Drägerverk AG, Lubeck, Germany) for inhalation. The surgical room had a controlled temperature set at 19 °C.

In each sheep, the metatarsal bone of one leg was shaved and disinfected with 40% ethanol and 0.5% chlorhexidine. After skin incision, the bone was exposed with periosteal elevator, and 4 implants were installed in the ventral metatarsal bone plate, consisting of approximately 4 mm of cortical bone, along the longitudinal direction.

Based on the drilling protocol and implant surface topography, four experimental groups were designed as showed in Table 1.

Each metatarsal bone received one implant per group. The sequence of the implantation for each metatarsal bone, according to the experimental group (A, B, C, D) was randomized by a computer-generated method, Microsoft^®^ excel (Version 15.30). The sequential implant osteotomies were free-hand prepared at approximately 20 mm inter-implant distance.

**Figure 1:**
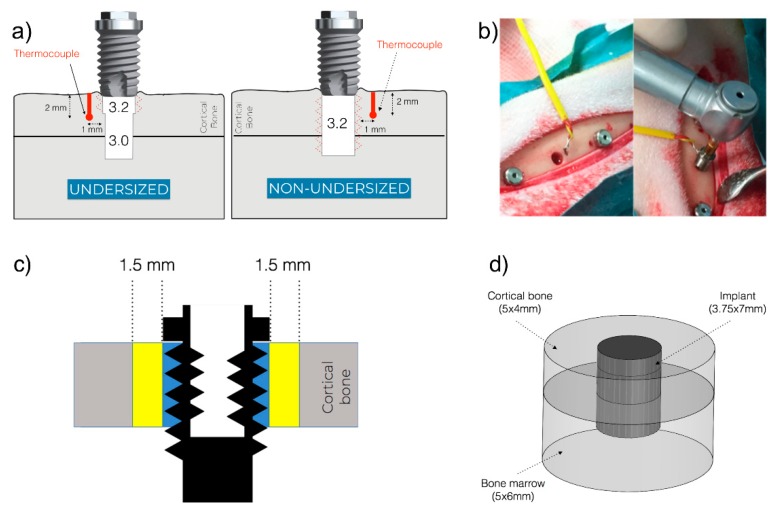
(**a**) Representation of the osteotomy preparation: the undersized drilling protocol on the left and non-undersized drilling protocol on the right. The osteotomy dimensions are depicted in the figures. The thermocouple site was prepared 2 mm deep into the cortical bone and 1 mm from the implant surface. (**b**) Overview of the surgical field with the thermocouple in position before implant installation (**left**) and during implant installation (**right**). (**c**) Representation of the regions of interest for histomorphometrical parameters. Bone-to-implant Contact (BIC) was calculated only for the cortical portion. Bone Area Fraction Occupancy (BAFO) was calculated in the blue part. Bone Area 1.5 (BA1.5) was calculated in the yellow part. (**d**) The Finite Element Model (FEM) was composed of three cylindrical elements: Cortical bone, bone marrow, and implant.

The surgical instrumentation sequence was performed following two different drilling protocols. In all groups, a 2.0 mm drill, 2.8 mm step drill, and 3.0 mm drill were used. Hereafter, the osteotomy was finalized based on the group (Figure 1a):

- Group A and C (undersized drilling protocol): 3.2 drill and tap drill were subsequently used for the preparation of the coronal 1.5 mm, in order to favor the engagement of the first threads during the implant installment.
- Group B and D (non-undersized drilling protocol): 3.2 drill and tap drill were subsequently used for the entire thickness of the cortical bone.

All drilling procedures were performed at 1200 rpm under abundant saline irrigation using a total of two new drill sets. Implant installation was carried out at 25 rpm without saline irrigation. Both the drilling procedure and implant installation were performed using a SA-310 W&H Elcomed implant unit (W&H, Burmoos, Austria). The insertion torque value (ITV) was recorded using the specific function and saved to a USB memory. Implants where ITV exceeded 80 Ncm during installation were manually installed by a manual torque wrench. Based on the ITV, three classes were distinguished: ITV ≤ 45 Ncm; 45 < ITV < 80 Ncm; ITV ≥ 80 Ncm. The flap was closed using a resorbable suture (Vicryl™, Ethicon^®^, Sommerville, NJ, USA) for the inner layer and non-absorbable suture for the external layer (Ethicon™, Ethicon^®^, Sommerville, NJ, USA). Morphine (0.1 mg/kg) was given intravenously every second hour during the operation and subcutaneously for the three first postoperative days.

### 2.3. Temperature Measurement

Intraosseous temperature was measured using the type-K thermocouple (Omega Engineering Limited, Manchester, UK) with a 0.5 mm tip, coupled to a HH12C handheld thermometer (Omega Engineering Limited, Manchester, UK). This dual-channel meter device is able to record the lower and maximum temperature with 2.5 measurements per second and a resolution of 0.1 °C. The tip of the thermocouple was inserted in a prepared drilled hole in the proximity of the implant osteotomy. More in details, the thermocouple site was prepared in a predetermined position in a proximal or distal site at 1 mm from the implant surface (Figure 1a) with the aid of a metal surgical template. To prepare the site, which had a diameter of 0.5 mm and a depth of 2 mm, a lance drill (Precision Drill, Nobel Biocare AB, Göteborg, Sweden) was used. To ensure the reproducibility of the hole depth, the drill was inserted until the marked line met the upper border of the template. Once the osteotomy was prepared, the thermocouple was inserted and kept the position until the thermometer showed a stable value (basal temperature). The installation of the implants was carried out approximately 1 min after the finalization of the drilling procedure. During the implant installation, the temperature was measured, and the maximum value was recorded as the maximum value (Figure 1b). The difference between the maximum and basal temperature for each site was calculated as the temperature change.

### 2.4. Preparation of Samples

After a healing period of five weeks, all animals were euthanized with an intravenous injection of a combination of 4000 mg embutramide, 538.4 mg mebezonium, and 87.8 mg tetracaine (T61, Intervet International, Unterschleißheim, Germany) and metatarsal bone blocks containing the implants were retrieved. Blocks were fixed in 4% formalin for seven days before dehydration by ascending concentration of ethanol, and embedded in light-curing methylmethacrylate (Technovit 7200 VLC, Heraeus Kulzer, Wehrheim, Germany) for undecalcified ground section procedures. The embedded bone samples with implant were sectioned parallel to the long axis at the center position of the implant using a diamond saw cutting machine (EXAKT 300, EXAKT Advanced Technologies GmbH, Norderstedt, Germany), and grinded and polished until a final section thickness of 30 μm (EXAKT 400CS, EXAKT Advanced Technologies GmbH, Norderstedt, Germany). The non-decalcified sections were stained in toluidine blue and pyronin G, and photographed using light microscopy with a digital imaging system (NanoZoomer S210, HAMAMATSU, Shizuoka, Japan).

### 2.5. Histomorphometric Analysis

Quantitative histomorphometry was performed considering the cortical layer only. All measurements were carried out with an image analysis software (Image J v.1.43u, National Institute of Health). The following variables were calculated:

- Bone-to-implant contact (BIC): On each side of the implant, the percentage of the bone in direct contact with the implant surface in the entire length of the implant placed in the bone was calculated. The mean value of the two sides was used for each implant.
- Bone Area Fraction Occupancy (BAFO): On each side of the implant, the percentage of the area within the implant threads occupied by visibly distinguishable bone was calculated. The mean value of the two sides was used for each implant.
- Bone Area Fraction Occupancy up to 1.5 mm (BA1.5): On each side of the implant a region of interest up to 1.5 mm from the implant outer diameter line was considered (Figure 1c). The percentage of the area occupied by visibly distinguishable bone was calculated. The mean value of the two sides was used for each implant.

### 2.6. Finite Element Model

In order to have a deeper understanding of the thermal behavior at the peri-implant bone, a Finite Element Model (FEM) was generated (Figure 1d). The model was composed of three cylindrical elements: Cortical bone (dimensions: 5 mm × 4 mm), bone marrow (dimensions: 5 mm × 6 mm), and implant (dimensions: 3.75 mm × 7 mm) designed by the CAD software (Solidworks Simulation 2011, Dassault Systèmes Solidworks, Waltham, MA, USA). Two different calorific values were set to the upper and bottom surface of the fixture related to the drilling hole with different diameters.

Steady conduction analysis was conducted by voxel-based Finite Element Analysis (FEA) software (VOXELCON2015, Quint, Fuchu, Japan). The thermal conductivity of cortical bone, bone marrow, and implant were set to 0.6 W/mK, 0.3 W/mK, and 20 W/mK, respectively [32,33]. The air temperature around those models was set to 19 °C.

The temperature change in the location of the thermocouple (2 mm depth from cortical bone surface and 1 mm away from the fixture surface) was approximated to 8 °C and 4 °C for the undersized drilling protocol groups and non-undersized drilling protocol groups, respectively. Until converging the calorific value, the steady conduction analysis was repeatedly conducted.

### 2.7. Statistical Methods

Categorical variables were reported as relative frequency, while continuous variables were reported as mean ± standard deviation after checking the normality of the distribution. Wilcoxon rank-sum test was used to test the influence of the drilling protocol and implant surface on temperature change.

Multiple regression models were used to evaluate the effect of the drilling protocol, implant surface, temperature change on the histomorphometric parameters (BIC, BAFO, BA1.5). *p*-values < 0.05 were considered statistically significant. The R statistical software package was used for the statistical evaluation and modelling (available at www.r-project.org/).

## 3. Results

### 3.1. ITV, General Healing and Temperature

**Figure 2:**
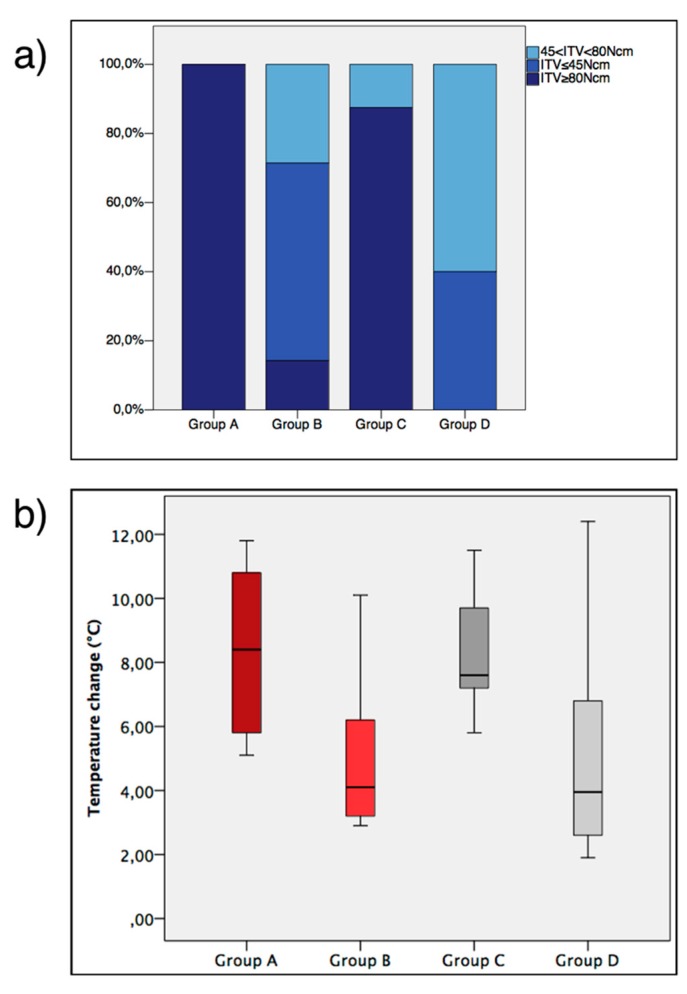
(**a**) Cumulative percentage of insertion torque value (ITV) classes divided per group. Note that all implants installed for group A had a ITV ≥ 80 Ncm. (**b**) Temperature change represented in box-plots.

All animals survived during surgical procedures and the experimental period. No implant was lost during the healing period. Signs of minor periosteal reaction were noted in three metatarsal bone samples. Such a response did not undermine the peri-implant bone healing and was limited to the periosteal area, which was not considered in this study. All implants were included in the statistical analysis. The relative distribution of the ITV class among the groups is displayed in Figure 2a.

The values for basal and maximum temperature are indicated in Table 2. Temperature change values are shown in Figure 2b. The temperature change was affected by the drilling protocol (*p* < 0.001), while it was not influenced by the surface topography (*p* = 0.879).

### 3.2. Histomorphometric Parameters

**Figure 3:**
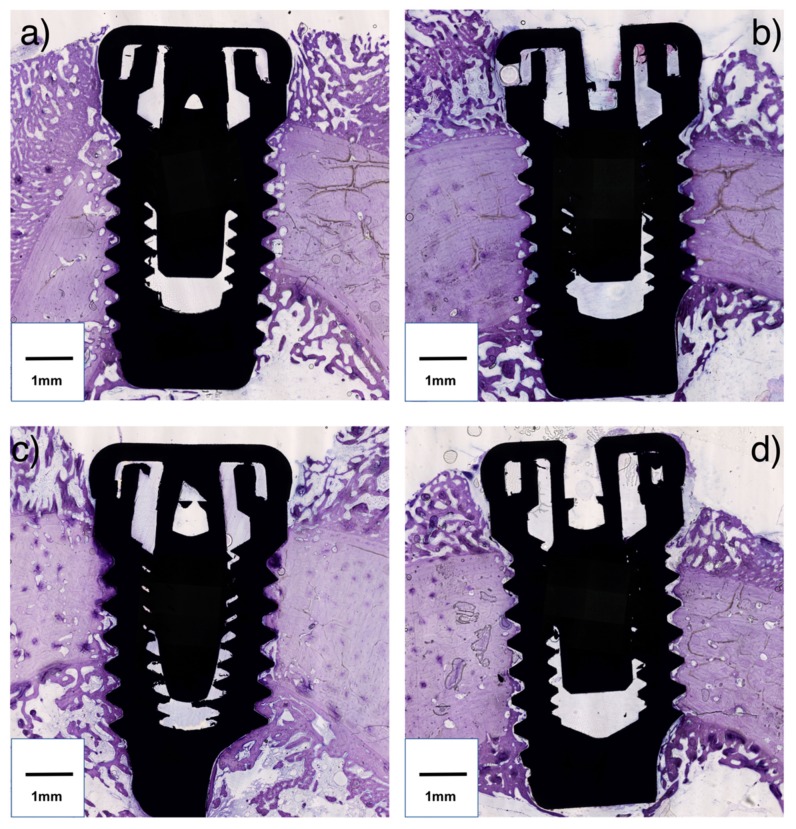
Histologic sections of the implant and peri-implant bone (original magnification 20×). Representations of group A, B, C, and D are depicted in (**a**), (**b**), (**c**), and (**d**), respectively.

Representation of the histologic sections are displayed in Figure 3.

**Figure 4:**
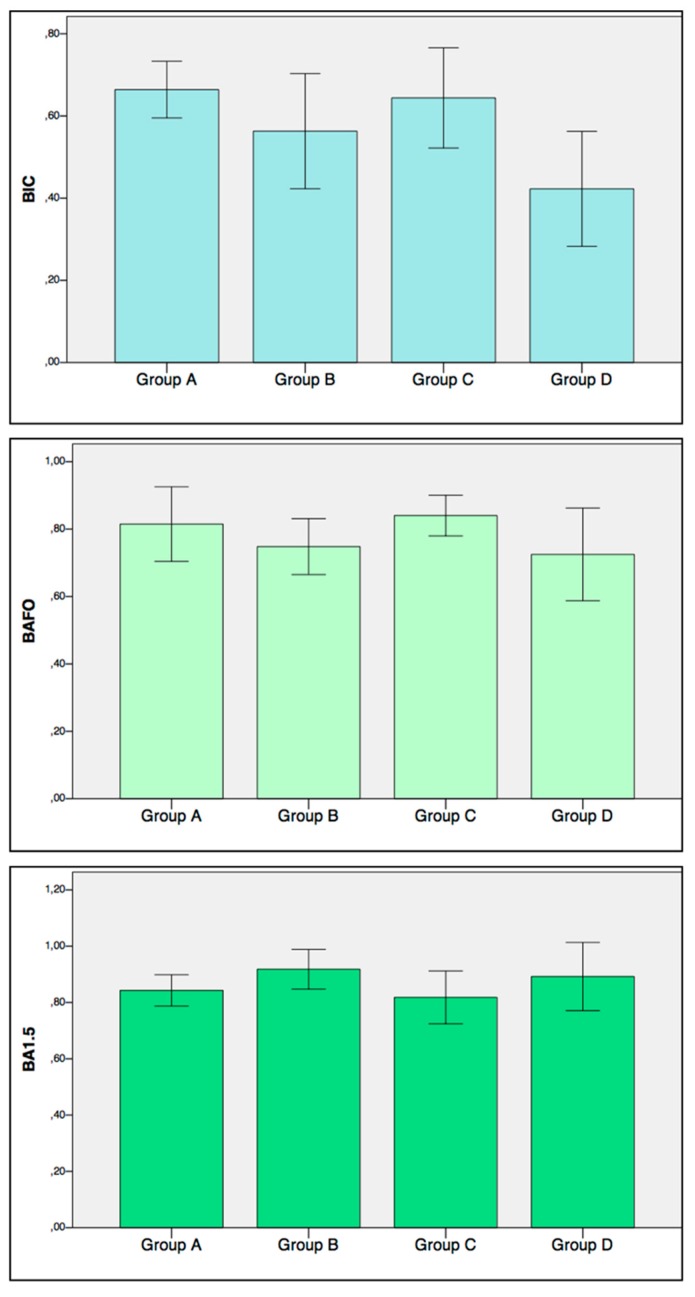
Bone to Implant Contact (**BIC**), Bone Area Fraction Occupancy (**BAFO**), and Bone Area Fraction Occupancy up to 1.5 mm (**BA1.5**) results represented as mean values. Error bars represent the standard deviation.

Mean values and standard deviation for BIC, BAFO, and BA1.5 are presented in Figure 4.

According to robust multiple regression analysis (Adjusted *R^2^*: 0.37, *p* = 0.0001), BIC was statistically significantly influenced by the implant surface (*p* = 0.01) and the drilling protocol (*p* = 0.0001). More specifically, BIC was increased with a moderately rough surface and undersized drilling protocol. BAFO was negatively affected by temperature increase (*p* = 0.01). Moreover BAFO was positively affected by the undersized drilling protocol (*p* = 0.0006) (Adjusted *R^2^*: 0.24, *p* = 0.005). BA1.5 was moderately affected by the undersized drilling protocol (*p* < 0.001) (Adjusted *R^2^*: 0.11, *p* = 0.06 for the model). No significant influence on BA1.5 was noted for the temperature change.

### 3.3. Finite Element Model

**Figure 5:**
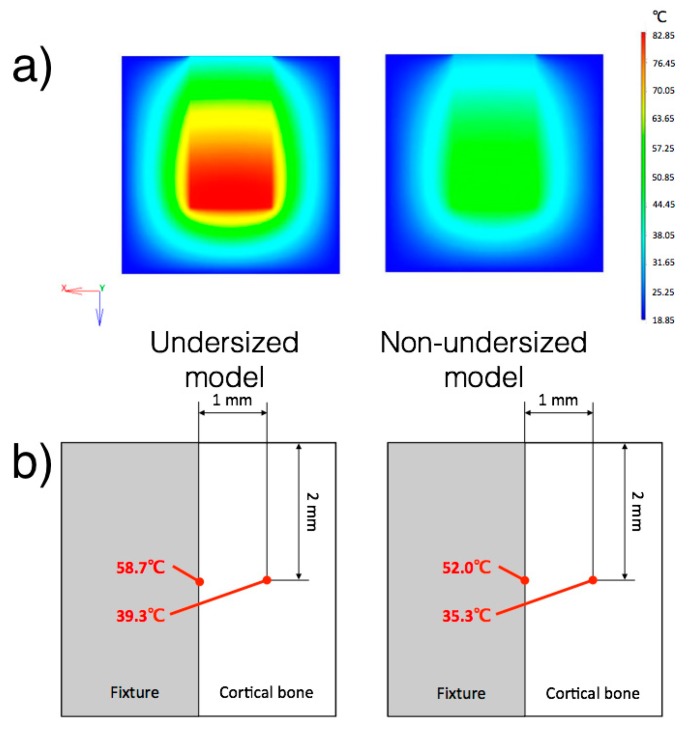
(**a**) The temperature distribution calculated on the Finite Element Model (FEM). The undersized drilling model and non-undersized model are displayed on the left and on the right, respectively. The temperature is displayed in °C. Note how the heat is poorly distributed around the implant surface. This means that the overheating risk is limited to the bone in the proximity to the implant. (**b**) Calculation of the bone temperature at the implant interface according to the FEM. This value was calculated by entering the value recorded with the thermocouple in the experimental part.

Temperature distribution at the center section is shown in Figure 5. The calorific value of the normal model was 30.8 W/m^3^. The calorific values of the upper fixture and bottom fixture were 30.8 W/m^3^ and 60.4 W/m^3^, respectively. The calorific value of fixture for the undersized drilling model showed 1.96 times greater than that for the non-undersized drilling model. The bone temperature at the interface with the implant surface was calculated for both models.

## 4. Discussion

In the present in vivo study, the intraosseous temperature was measured during dental implant installation into cortical bone, following two different drilling protocols and with two different implant surfaces. In addition, histomorphometric parameters of osseointegration were evaluated in relation to the bone temperature recorded during the implant installation and a computational model was created to examine the thermal distribution. To the knowledge of the authors, the current investigation represents the first in vivo experiment with this setup.

### 4.1. Bone Temperature

In the present study, it was discovered that the intraosseous temperature during implant installation was influenced by the drilling protocol. During implant installation, a certain amount of energy is also dissipated into heat [25]. As previous in vitro studies have observed, the rotational torque is positively related to bone heating during implant installation [24,34]. Accordingly, in this study, the installation of implants into undersized sites developed a great friction resistance, resulting in both in a higher ITV (Figure 3) and temperature increase, compared with non-undersized osteotomies. Specifically, the undersized drilling protocol groups resulted in a median increase of temperature of approximately 8 °C, while in the non-undersized drilling protocol groups, it was approximately 4 °C. The maximum recorded temperature of 45.3 °C exceeded the limit of cell damage, which is 45 °C according to Ludewig [35], but it was lower than the critical value for bone necrosis, which is 50 °C according to Lundskog [36]. In the early 1980s, Eriksson, whose doctoral thesis greatly contributed to the knowledge on bone tissue regeneration, stated that the threshold level for bone survival was 47 °C for 1 min [37]. However, the temperature recorded in the present study was detected at 1 mm from the implant surface. To have a better understanding of the temperature behavior in the proximity of the implant, a FEM was designed. It was estimated that the temperature at the bone-implant interface for the undersized groups and non-undersized groups reached 58.7 °C and 52.0 °C respectively (Figure 5). Thus, according to the FEM calculation, the installation of the implant caused a frictional heat over the critical temperature for bone injury at the bone-implant interface. One may expect that implants installed with an undersized drilling protocol would create a major extent of tissue damage. Still, such an overheating condition is restricted to the proximity of the implant, as shown by the model. Since the temperature change was affected by the drilling protocol (*p* < 0.001), but was not influenced by the surface topography (*p* = 0.879), the surface characteristics (parameters Sa and Sdr) were not included in the design of the FEM.

Such results are confirmed by histomorphometry. In effect, it was demonstrated that the temperature generated during implant installation has a tangible biologic impact, since the amount of bone between the threads, namely BAFO, is negatively affected by the temperature increase. This finding is in accordance with Eriksson’s study, in which they observed a loss of 10% of bone tissue after 30 days when a rabbit tibia was heated to 47 °C for 1 min [22]. This study supports the conclusion that the portion of the bone in proximity with the implant surface might be the most sensitive to heating at the implant installation. Moreover, based on the FEM analysis and histomorphometric results, this heating might induce bone damage if the undersized drilling protocol is applied. The influence of the heat generated by the drilling procedures was likely to be excluded since one minute elapsed before the implant installation. Previous research indicated that the bone returned to baseline temperature after approximately 30 s [38].

Nevertheless, due to the low thermal conductivity properties of cortical bone, we could expect a low grade of heat distribution through the bone [39]. Thus, the risk of bone overheating is limited to the bone in the proximity to the bone-to-implant interface.

It has to be said that Eriksson observed how bone cells are susceptible to the exposure time, other than peak temperature [40]. In the present experiment, the maximum temperature endured for a few seconds, then it gradually descended. However, the actual temperature/time curve was not recorded, representing a limitation of the study.

### 4.2. Drilling Protocol

The present findings showed that an undersized drilling protocol per se might not be detrimental to osseointegration for the Brånemark implant. In a previous study by the authors, implants were inserted in sheep mandible according to two different drilling techniques [41]. The results showed that implants inserted into an undersized osteotomy caused tissue damage to the peri-implant bone. In particular, large remodeling cavities with resorption activity were noted, and a lower amount of bone was identified up to 1.5 mm distance from the implant, both from histomorphometric µ-CT analysis. In addition, the drilling protocol seemed not to influence the amount of total BIC. Such findings are partially confirmed by the present results, since the amount of bone up to 1.5 mm from implant surface (BA1.5), was negatively influenced by the undersized drilling protocol. An explanation could be that the bone compression during the implant installation in a tight osteotomy would trigger a remodeling process at a distance from the implant interface. On the other hand, the temperature change does not influence this parameter, since the thermal conductivity of the bone would prevent the heat from being transferred at such a distance from the implant.

Compared with the previous sheep study [41], a number of differences were noted. In the former study, an implant with a micro-threaded neck was used, while the Brånemark type implant was utilized in the present investigation. A previous FEA study on the press-fit phenomena at the implant insertion, [42] affirmed that the micro-thread portion, induced more relevant strains compared to the situation without microthreads. Thus, one could expect greater bone damage after undersized drilling in such a scenario. On the contrary, the undersized drilling protocol positively influenced the amount of bone in close proximity to the implant, i.e., BIC and BAFO, in the present study. It could be assumed that a large portion of the bone in contact with the implant might be the original bone that was forced to the implant surface during the implant installation and still not removed. This finding was observed in several previous studies, which used a similar implant design [5,43,44]. In normal conditions, such tissue will be gradually resorbed with time and eventually substituted with vital bone, according to the remodeling process [45].

### 4.3. Surface

An interesting finding of the present study was that the type of implant surface did not influence the bone temperature. It could be expected that a moderately rough surface could increase the friction and thereby the heat [46,47]. However, no differences were noticed in the temperature increase between moderately rough and turned surfaced implants. According to the results, a moderately rough surface had a positive effect on the amount of bone in contact with the implant. This finding confirms that such surface topography is able to promote the osteoconductive properties of the implant, compared to turned surfaced implants, as reported by animal and human histologic reports by Ivanoff et al. and Zechner et al. [28,48].

### 4.4. Clinical Applications and Limitations

The study represents one of the first attempts to study the implant insertion temperature in an in vivo setting. Results demonstrated that an undersized drilling protocol causes an increase of intraosseous temperature during implant seating. A temperature increase negatively affected the amount of peri-implant bone. Thus, it may be suggested to reduce the friction overheating during implant installation. This would include decreasing the rotational speed [49], the use of the self-tapping implant design [24], the use of irrigation during implant installation, and the selection of the proper drilling protocol based on the implant design and the bone quality.

Considering the limitations in the present study, the record of the temperature was limited to the peak value during each insertion. Further studies are needed to evaluate the exact duration of the heat. In addition, from the present study, we cannot confirm whether the increase of the temperature caused tissue necrosis, since no specific stain for cell metabolism and tissue turnover was used. Moreover, the relationship between the compression and the heat, following undersized drilling was not explored. Future studies should be designed in order to indicate whether there is a predominant factor in the generation of tissue damage in the proximity of the implant. Finally, it must be stated that the sheep model, which has been broadly used in dental implant research, presents similarities and differences compared to human bone [50]. The thickness and density of cortical bone may approximate clinical scenarios, such as encountered in the mandible. In addition, sheep bone turnover resembles bone processes in humans, even though it is slightly more rapid. The healing period of five weeks was selected according to our previous research [41], since the influence of the surgical protocol is more evident at this stage in the peri-implant bone. However, the metatarsal bone, while it represents an accessible and convenient substrate for orthopedic and dental implant research, presents quite large anatomical and physiological differences compared with the human jaw, and it may display slightly divergent thermal properties. Therefore the present findings must be taken with reasonable caution.

## 5. Conclusions

Within the limitations of the present study, it was shown that different drilling protocol for the Brånemark implant system affects both the intraosseous temperature during implant installation and the peri-implant bone healing. The undersized drilling protocol provokes a greater increase of bone temperature in the proximity of the implant compared with non-undersized drilling. The temperature at the bone-implant interface may exceed the critical value for thermal necrosis, and it may have negative effects on peri-implant bone healing. The present results indicate that undersized drilling increases the amount of bone in the proximity of the implant, but it has a negative impact on the bone area far from the implant surface. The moderately rough surface does not influence the bone temperature, while it increased the bone attached to the implant. Further studies are needed to confirm the present results and to deeply investigate the thermal behavior and the biologic effect of peri-implant bone overheating during implant installation and to provide guidelines on the clinical decisions for the proper drilling protocol, based on the bone quality and implant design.

## Figures and Tables

**Table 1:** Description of the experimental groups.

	Drilling Protocol	Implant Surface
Group A (n = 10)	Undersized	Moderately rough
Group B (n = 10)	Non-undersized	Moderately rough
Group C (n = 10)	Undersized	Turned
Group D (n = 10)	Non-undersized	Turned

**Table 2:** Basal temperature and maximum temperature for the different groups are shown in °C. SD: Standard deviation; Max: maximum value; Min: minimum value.

	Basal Temperature				Maximum Temperature			
	Mean	SD	Max	Min	Mean	SD	Max	Min
Group A	31.3	2.3	34.5	28.4	39.6	3.3	45.3	34.7
Group B	31.0	3.4	34.7	23.7	36.0	3.4	41.5	28.6
Group C	31.0	3.3	34.0	23.6	39.1	3.7	44.0	33.4
Group D	31.3	2.3	34.2	27.5	36.4	1.8	39.9	33.6
